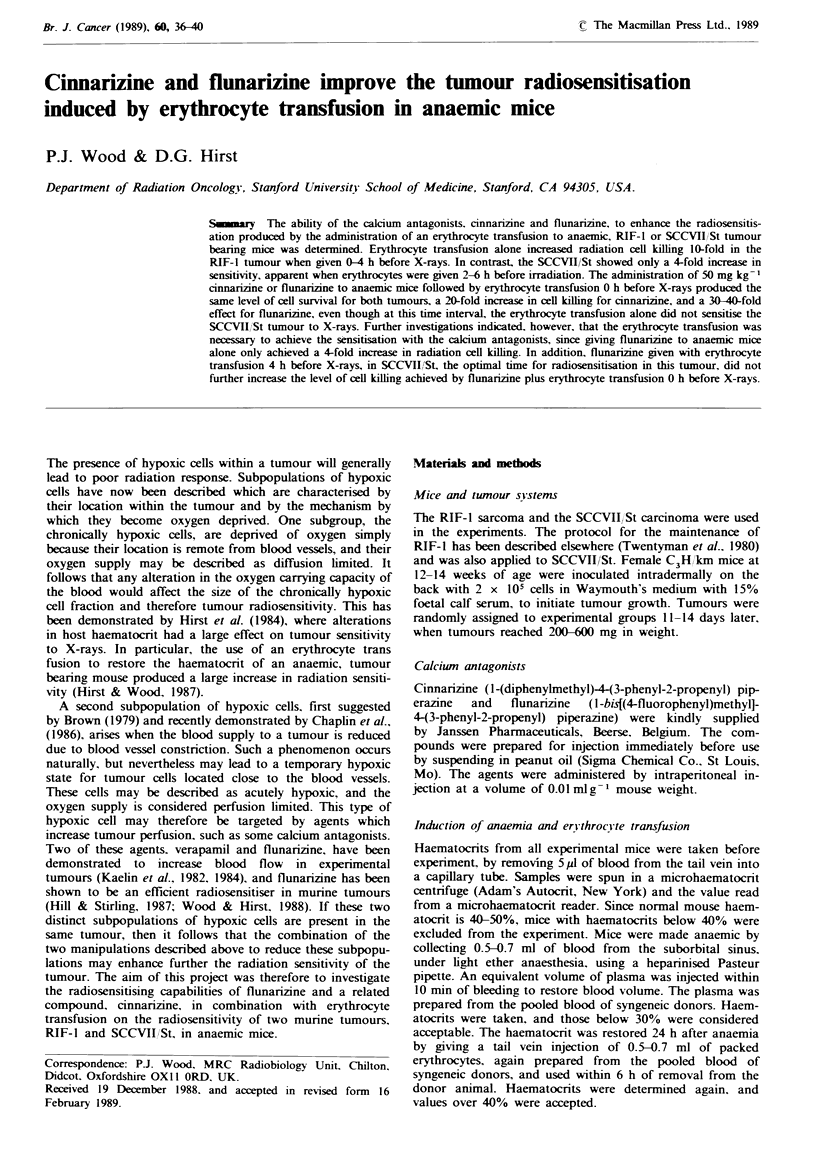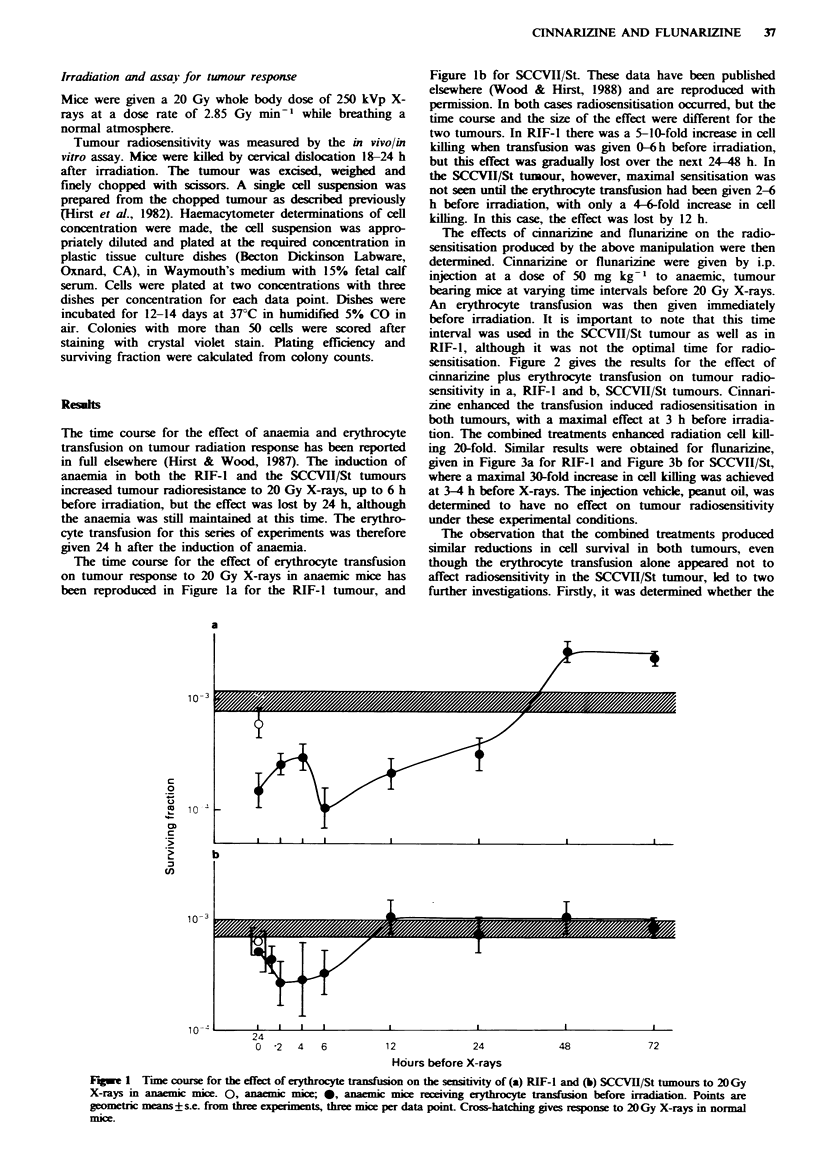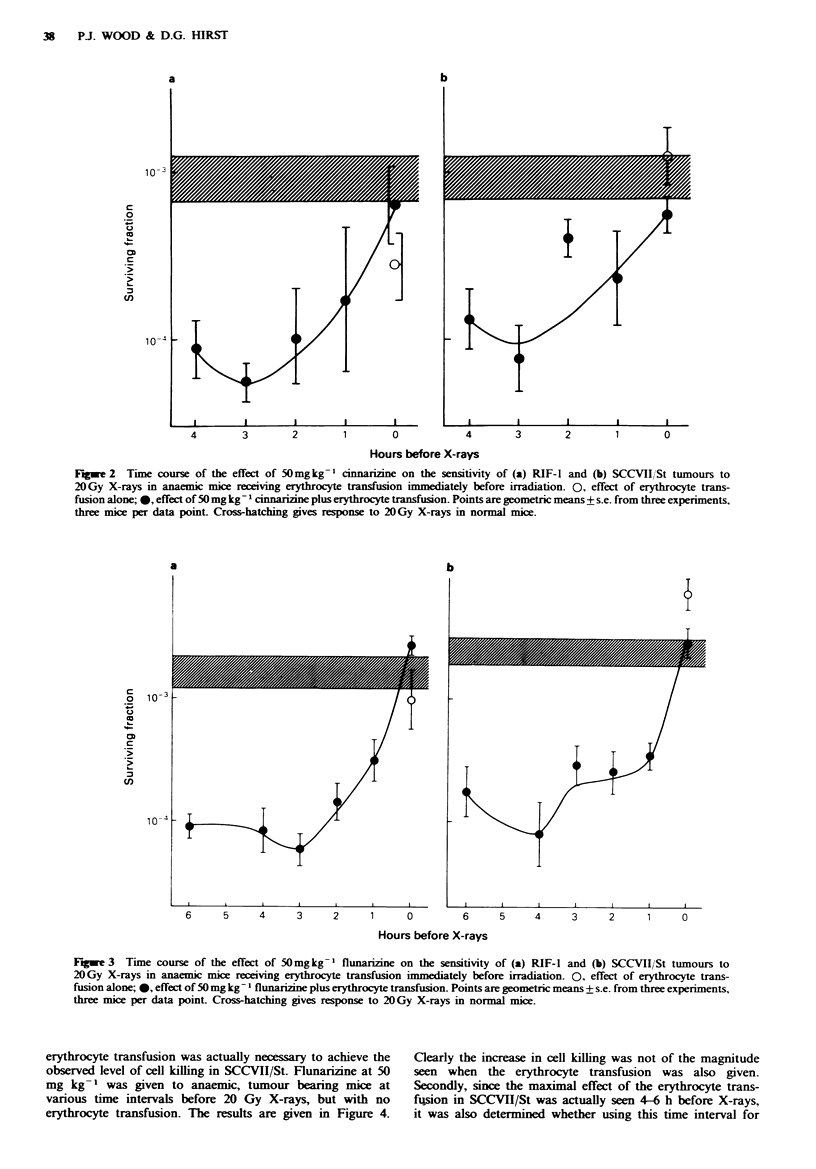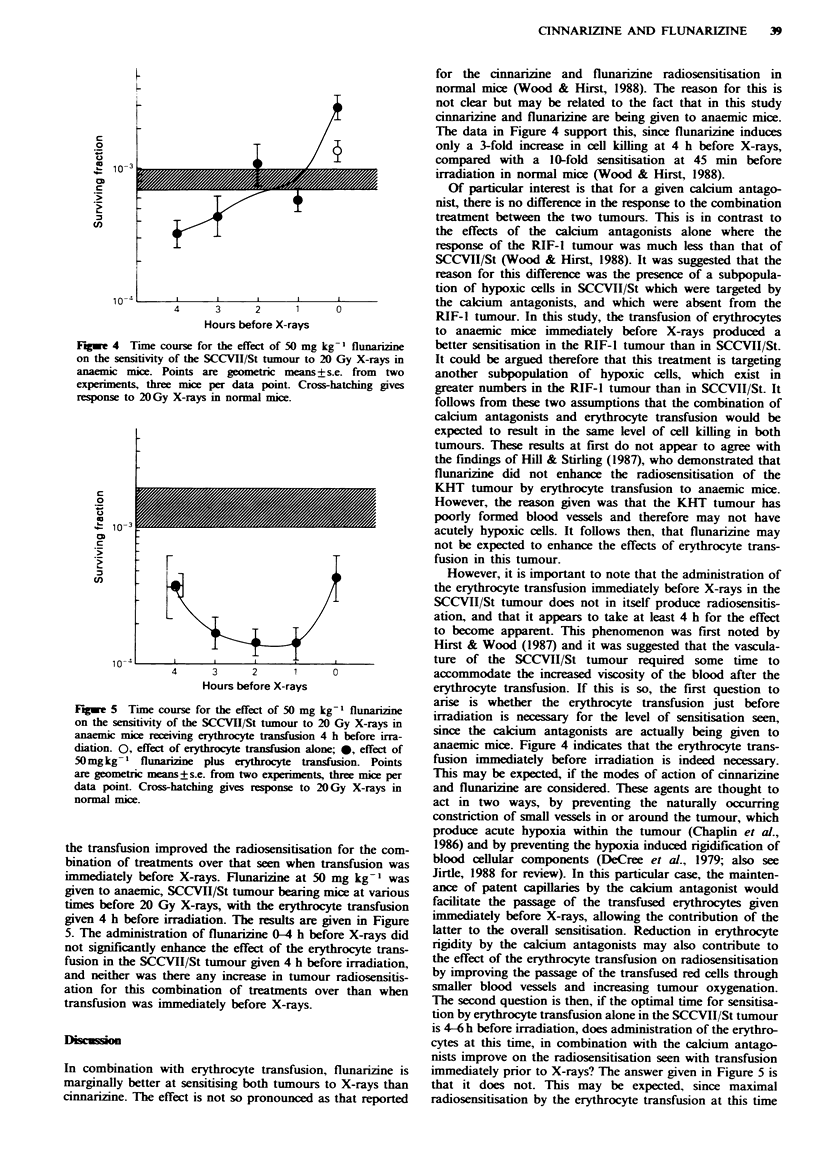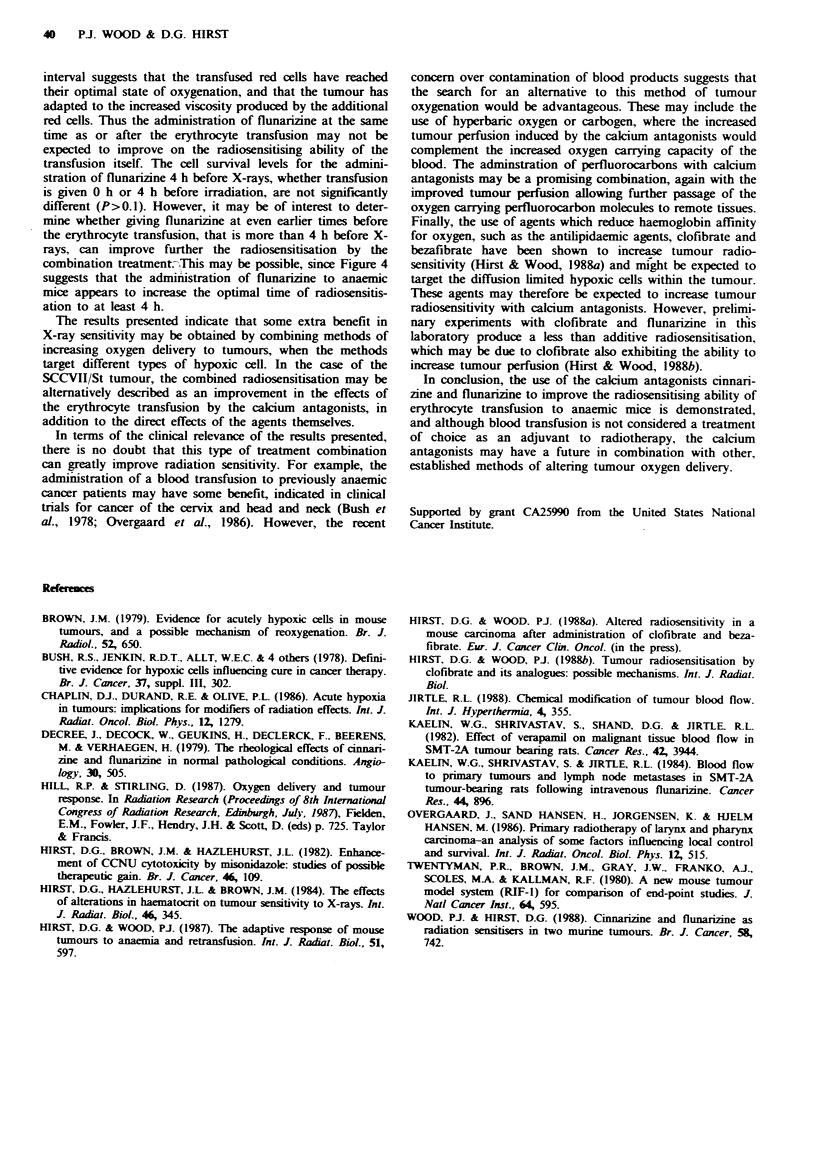# Cinnarizine and flunarizine improve the tumour radiosensitisation induced by erythrocyte transfusion in anaemic mice.

**DOI:** 10.1038/bjc.1989.215

**Published:** 1989-07

**Authors:** P. J. Wood, D. G. Hirst

**Affiliations:** Department of Radiation Oncology, Stanford University School of Medicine, CA 94305.

## Abstract

The ability of the calcium antagonists, cinnarizine and flunarizine, to enhance the radiosensitisation produced by the administration of an erythrocyte transfusion to anaemic, RIF-1 or SCCVII/St tumour bearing mice was determined. Erythrocyte transfusion alone increased radiation cell killing 10-fold in the RIF-1 tumour when given 0-4 h before X-rays. In contrast, the SCCVII/St showed only a 4-fold increase in sensitivity, apparent when erythrocytes were given 2-6 h before irradiation. The administration of 50 mg kg-1 cinnarizine or flunarizine to anaemic mice followed by erythrocyte transfusion 0 h before X-rays produced the same level of cell survival for both tumours, a 20-fold increase in cell killing for cinnarizine, and a 30-40-fold effect for flunarizine, even though at this time interval, the erythrocyte transfusion alone did not sensitise the SCCVII/St tumour to X-rays. Further investigations indicated, however, that the erythrocyte transfusion was necessary to achieve the sensitisation with the calcium antagonists, since giving flunarizine to anaemic mice alone only achieved a 4-fold increase in radiation cell killing. In addition, flunarizine given with erythrocyte transfusion 4 h before X-rays, in SCCVII/St, the optimal time for radiosensitisation in this tumour, did not further increase the level of cell killing achieved by flunarizine plus erythrocyte transfusion 0 h before X-rays.


Br. J. Cancer (1989), 60, 36–40 © The Macmillan Press Ltd., 1989

### Cinnarizine and flunarizine improve the tumour radiosensitisation induced by erythrocyte transfusion in anaemic mice

P.J. Wood & D.G. Hirst

*Department of Radiation Oncology, Stanford University School of Medicine, Stanford, CA 94305, USA.*

**Summary** The ability of the calcium antagonists, cinnarizine and flunarizine, to enhance the radiosensitisation produced by the administration of an erythrocyte transfusion to anaemic, RIF-1 or SCCVII/St tumour bearing mice was determined. Erythrocyte transfusion alone increased radiation cell killing 10-fold in the RIF-1 tumour when given 0–4 h before X-rays. In contrast, the SCCVII/St showed only a 4-fold increase in sensitivity, apparent when erythrocytes were given 2–6 h before irradiation. The administration of 50 mg $kg^{-1}$ cinnarizine or flunarizine to anaemic mice followed by erythrocyte transfusion 0 h before X-rays produced the same level of cell survival for both tumours, a 20-fold increase in cell killing for cinnarizine, and a 30–40-fold effect for flunarizine, even though at this time interval, the erythrocyte transfusion alone did not sensitise the SCCVII/St tumour to X-rays. Further investigations indicated, however, that the erythrocyte transfusion was necessary to achieve the sensitisation with the calcium antagonists, since giving flunarizine to anaemic mice alone only achieved a 4-fold increase in radiation cell killing. In addition, flunarizine given with erythrocyte transfusion 4 h before X-rays, in SCCVII/St, the optimal time for radiosensitisation in this tumour, did not further increase the level of cell killing achieved by flunarizine plus erythrocyte transfusion 0 h before X-rays.

The presence of hypoxic cells within a tumour will generally lead to poor radiation response. Subpopulations of hypoxic cells have now been described which are characterised by their location within the tumour and by the mechanism by which they become oxygen deprived. One subgroup, the chronically hypoxic cells, are deprived of oxygen simply because their location is remote from blood vessels, and their oxygen supply may be described as diffusion limited. It follows that any alteration in the oxygen carrying capacity of the blood would affect the size of the chronically hypoxic cell fraction and therefore tumour radiosensitivity. This has been demonstrated by Hirst *et al.* (1984), where alterations in host haematocrit had a large effect on tumour sensitivity to X-rays. In particular, the use of an erythrocyte trans fusion to restore the haematocrit of an anaemic, tumour bearing mouse produced a large increase in radiation sensitivity (Hirst & Wood, 1987).

A second subpopulation of hypoxic cells, first suggested by Brown (1979) and recently demonstrated by Chaplin *et al.*, (1986), arises when the blood supply to a tumour is reduced due to blood vessel constriction. Such a phenomenon occurs naturally, but nevertheless may lead to a temporary hypoxic state for tumour cells located close to the blood vessels. These cells may be described as acutely hypoxic, and the oxygen supply is considered perfusion limited. This type of hypoxic cell may therefore be targeted by agents which increase tumour perfusion, such as some calcium antagonists. Two of these agents, verapamil and flunarizine, have been demonstrated to increase blood flow in experimental tumours (Kaelin *et al.*, 1982, 1984), and flunarizine has been shown to be an efficient radiosensitiser in murine tumours (Hill & Stirling, 1987; Wood & Hirst, 1988). If these two distinct subpopulations of hypoxic cells are present in the same tumour, then it follows that the combination of the two manipulations described above to reduce these subpopulations may enhance further the radiation sensitivity of the tumour. The aim of this project was therefore to investigate the radiosensitising capabilities of flunarizine and a related compound, cinnarizine, in combination with erythrocyte transfusion on the radiosensitivity of two murine tumours, RIF-1 and SCCVII/St, in anaemic mice.

Correspondence: P.J. Wood, MRC Radiobiology Unit, Chilton, Didcot, Oxfordshire OX11 0RD, UK.
Received 19 December 1988, and accepted in revised form 16 February 1989.

### Materials and methods

### *Mice and tumour systems*

The RIF-1 sarcoma and the SCCVII/St carcinoma were used in the experiments. The protocol for the maintenance of RIF-1 has been described elsewhere (Twentyman *et al.*, 1980) and was also applied to SCCVII/St. Female $C_3H$/km mice at 12–14 weeks of age were inoculated intradermally on the back with 2 × $10^5$ cells in Waymouth's medium with 15% foetal calf serum, to initiate tumour growth. Tumours were randomly assigned to experimental groups 11–14 days later, when tumours reached 200–600 mg in weight.

### *Calcium antagonists*

Cinnarizine (1-(diphenylmethyl)-4-(3-phenyl-2-propenyl) piperazine and flunarizine (1-*bis*[(4-fluorophenyl)methyl]-4-(3-phenyl-2-propenyl) piperazine) were kindly supplied by Janssen Pharmaceuticals, Beerse, Belgium. The compounds were prepared for injection immediately before use by suspending in peanut oil (Sigma Chemical Co., St Louis, Mo). The agents were administered by intraperitoneal injection at a volume of 0.01 ml $g^{-1}$ mouse weight.

### *Induction of anaemia and erythrocyte transfusion*

Haematocrits from all experimental mice were taken before experiment, by removing 5 $\mu$l of blood from the tail vein into a capillary tube. Samples were spun in a microhaematocrit centrifuge (Adam's Autocrit, New York) and the value read from a microhaematocrit reader. Since normal mouse haematocrit is 40–50%, mice with haematocrits below 40% were excluded from the experiment. Mice were made anaemic by collecting 0.5–0.7 ml of blood from the suborbital sinus, under light ether anaesthesia, using a heparinised Pasteur pipette. An equivalent volume of plasma was injected within 10 min of bleeding to restore blood volume. The plasma was prepared from the pooled blood of syngeneic donors. Haematocrits were taken, and those below 30% were considered acceptable. The haematocrit was restored 24 h after anaemia by giving a tail vein injection of 0.5–0.7 ml of packed erythrocytes, again prepared from the pooled blood of syngeneic donors, and used within 6 h of removal from the donor animal. Haematocrits were determined again, and values over 40% were accepted.

*Irradiation and assay for tumour response*

Mice were given a 20 Gy whole body dose of 250 kVp X-rays at a dose rate of 2.85 Gy $min^{-1}$ while breathing a normal atmosphere.

Tumour radiosensitivity was measured by the *in vivo/in vitro* assay. Mice were killed by cervical dislocation 18–24 h after irradiation. The tumour was excised, weighed and finely chopped with scissors. A single cell suspension was prepared from the chopped tumour as described previously (Hirst *et al.*, 1982). Haemacytometer determinations of cell concentration were made, the cell suspension was appropriately diluted and plated at the required concentration in plastic tissue culture dishes (Becton Dickinson Labware, Oxnard, CA), in Waymouth's medium with 15% fetal calf serum. Cells were plated at two concentrations with three dishes per concentration for each data point. Dishes were incubated for 12–14 days at 37°C in humidified 5% CO in air. Colonies with more than 50 cells were scored after staining with crystal violet stain. Plating efficiency and surviving fraction were calculated from colony counts.

### Results

The time course for the effect of anaemia and erythrocyte transfusion on tumour radiation response has been reported in full elsewhere (Hirst & Wood, 1987). The induction of anaemia in both the RIF-1 and the SCCVII/St tumours increased tumour radioresistance to 20 Gy X-rays, up to 6 h before irradiation, but the effect was lost by 24 h, although the anaemia was still maintained at this time. The erythrocyte transfusion for this series of experiments was therefore given 24 h after the induction of anaemia.

The time course for the effect of erythrocyte transfusion on tumour response to 20 Gy X-rays in anaemic mice has been reproduced in Figure 1a for the RIF-1 tumour, and Figure 1b for SCCVII/St. These data have been published elsewhere (Wood & Hirst, 1988) and are reproduced with permission. In both cases radiosensitisation occurred, but the time course and the size of the effect were different for the two tumours. In RIF-1 there was a 5–10-fold increase in cell killing when transfusion was given 0–6 h before irradiation, but this effect was gradually lost over the next 24–48 h. In the SCCVII/St tumour, however, maximal sensitisation was not seen until the erythrocyte transfusion had been given 2–6 h before irradiation, with only a 4–6-fold increase in cell killing. In this case, the effect was lost by 12 h.

The effects of cinnarizine and flunarizine on the radiosensitisation produced by the above manipulation were then determined. Cinnarizine or flunarizine were given by i.p. injection at a dose of 50 mg $kg^{-1}$ to anaemic, tumour bearing mice at varying time intervals before 20 Gy X-rays. An erythrocyte transfusion was then given immediately before irradiation. It is important to note that this time interval was used in the SCCVII/St tumour as well as in RIF-1, although it was not the optimal time for radiosensitisation. Figure 2 gives the results for the effect of cinnarizine plus erythrocyte transfusion on tumour radiosensitivity in a, RIF-1 and b, SCCVII/St tumours. Cinnarizine enhanced the transfusion induced radiosensitisation in both tumours, with a maximal effect at 3 h before irradiation. The combined treatments enhanced radiation cell killing 20-fold. Similar results were obtained for flunarizine, given in Figure 3a for RIF-1 and Figure 3b for SCCVII/St, where a maximal 30-fold increase in cell killing was achieved at 3–4 h before X-rays. The injection vehicle, peanut oil, was determined to have no effect on tumour radiosensitivity under these experimental conditions.

The observation that the combined treatments produced similar reductions in cell survival in both tumours, even though the erythrocyte transfusion alone appeared not to affect radiosensitivity in the SCCVII/St tumour, led to two further investigations. Firstly, it was determined whether the

**Figure 1** Time course for the effect of erythrocyte transfusion on the sensitivity of (**a**) RIF-1 and (**b**) SCCVII/St tumours to 20 Gy X-rays in anaemic mice. O, anaemic mice; ●, anaemic mice receiving erythrocyte transfusion before irradiation. Points are geometric means ± s.e. from three experiments, three mice per data point. Cross-hatching gives response to 20 Gy X-rays in normal mice.

**Figure 2** Time course of the effect of $50\,mg\,kg^{-1}$ cinnarizine on the sensitivity of (**a**) RIF-1 and (**b**) SCCVII/St tumours to 20 Gy X-rays in anaemic mice receiving erythrocyte transfusion immediately before irradiation. ○, effect of erythrocyte transfusion alone; ●, effect of $50\,mg\,kg^{-1}$ cinnarizine plus erythrocyte transfusion. Points are geometric means ± s.e. from three experiments, three mice per data point. Cross-hatching gives response to 20 Gy X-rays in normal mice.

**Figure 3** Time course of the effect of $50\,mg\,kg^{-1}$ flunarizine on the sensitivity of (**a**) RIF-1 and (**b**) SCCVII/St tumours to 20 Gy X-rays in anaemic mice receiving erythrocyte transfusion immediately before irradiation. ○, effect of erythrocyte transfusion alone; ●, effect of $50\,mg\,kg^{-1}$ flunarizine plus erythrocyte transfusion. Points are geometric means ± s.e. from three experiments, three mice per data point. Cross-hatching gives response to 20 Gy X-rays in normal mice.

erythrocyte transfusion was actually necessary to achieve the observed level of cell killing in SCCVII/St. Flunarizine at 50 $mg\ kg^{-1}$ was given to anaemic, tumour bearing mice at various time intervals before 20 Gy X-rays, but with no erythrocyte transfusion. The results are given in Figure 4. Clearly the increase in cell killing was not of the magnitude seen when the erythrocyte transfusion was also given. Secondly, since the maximal effect of the erythrocyte transfusion in SCCVII/St was actually seen 4–6 h before X-rays, it was also determined whether using this time interval for

**Figure 4** Time course for the effect of 50 mg $kg^{-1}$ flunarizine on the sensitivity of the SCCVII/St tumour to 20 Gy X-rays in anaemic mice. Points are geometric means ± s.e. from two experiments, three mice per data point. Cross-hatching gives response to 20 Gy X-rays in normal mice.

**Figure 5** Time course for the effect of 50 mg $kg^{-1}$ flunarizine on the sensitivity of the SCCVII/St tumour to 20 Gy X-rays in anaemic mice receiving erythrocyte transfusion 4 h before irradiation. ○, effect of erythrocyte transfusion alone; ●, effect of 50 mg $kg^{-1}$ flunarizine plus erythrocyte transfusion. Points are geometric means ± s.e. from two experiments, three mice per data point. Cross-hatching gives response to 20 Gy X-rays in normal mice.

the transfusion improved the radiosensitisation for the combination of treatments over that seen when transfusion was immediately before X-rays. Flunarizine at 50 mg $kg^{-1}$ was given to anaemic, SCCVII/St tumour bearing mice at various times before 20 Gy X-rays, with the erythrocyte transfusion given 4 h before irradiation. The results are given in Figure 5. The administration of flunarizine 0–4 h before X-rays did not significantly enhance the effect of the erythrocyte transfusion in the SCCVII/St tumour given 4 h before irradiation, and neither was there any increase in tumour radiosensitisation for this combination of treatments over than when transfusion was immediately before X-rays.

### Discussion

In combination with erythrocyte transfusion, flunarizine is marginally better at sensitising both tumours to X-rays than cinnarizine. The effect is not so pronounced as that reported for the cinnarizine and flunarizine radiosensitisation in normal mice (Wood & Hirst, 1988). The reason for this is not clear but may be related to the fact that in this study cinnarizine and flunarizine are being given to anaemic mice. The data in Figure 4 support this, since flunarizine induces only a 3-fold increase in cell killing at 4 h before X-rays, compared with a 10-fold sensitisation at 45 min before irradiation in normal mice (Wood & Hirst, 1988).

Of particular interest is that for a given calcium antagonist, there is no difference in the response to the combination treatment between the two tumours. This is in contrast to the effects of the calcium antagonists alone where the response of the RIF-1 tumour was much less than that of SCCVII/St (Wood & Hirst, 1988). It was suggested that the reason for this difference was the presence of a subpopulation of hypoxic cells in SCCVII/St which were targeted by the calcium antagonists, and which were absent from the RIF-1 tumour. In this study, the transfusion of erythrocytes to anaemic mice immediately before X-rays produced a better sensitisation in the RIF-1 tumour than in SCCVII/St. It could be argued therefore that this treatment is targeting another subpopulation of hypoxic cells, which exist in greater numbers in the RIF-1 tumour than in SCCVII/St. It follows from these two assumptions that the combination of calcium antagonists and erythrocyte transfusion would be expected to result in the same level of cell killing in both tumours. These results at first do not appear to agree with the findings of Hill & Stirling (1987), who demonstrated that flunarizine did not enhance the radiosensitisation of the KHT tumour by erythrocyte transfusion to anaemic mice. However, the reason given was that the KHT tumour has poorly formed blood vessels and therefore may not have acutely hypoxic cells. It follows then, that flunarizine may not be expected to enhance the effects of erythrocyte transfusion in this tumour.

However, it is important to note that the administration of the erythrocyte transfusion immediately before X-rays in the SCCVII/St tumour does not in itself produce radiosensitisation, and that it appears to take at least 4 h for the effect to become apparent. This phenomenon was first noted by Hirst & Wood (1987) and it was suggested that the vasculature of the SCCVII/St tumour required some time to accommodate the increased viscosity of the blood after the erythrocyte transfusion. If this is so, the first question to arise is whether the erythrocyte transfusion just before irradiation is necessary for the level of sensitisation seen, since the calcium antagonists are actually being given to anaemic mice. Figure 4 indicates that the erythrocyte transfusion immediately before irradiation is indeed necessary. This may be expected, if the modes of action of cinnarizine and flunarizine are considered. These agents are thought to act in two ways, by preventing the naturally occurring constriction of small vessels in or around the tumour, which produce acute hypoxia within the tumour (Chaplin *et al*., 1986) and by preventing the hypoxia induced rigidification of blood cellular components (DeCree *et al*., 1979; also see Jirtle, 1988 for review). In this particular case, the maintenance of patent capillaries by the calcium antagonist would facilitate the passage of the transfused erythrocytes given immediately before X-rays, allowing the contribution of the latter to the overall sensitisation. Reduction in erythrocyte rigidity by the calcium antagonists may also contribute to the effect of the erythrocyte transfusion on radiosensitisation by improving the passage of the transfused red cells through smaller blood vessels and increasing tumour oxygenation. The second question is then, if the optimal time for sensitisation by erythrocyte transfusion alone in the SCCVII/St tumour is 4–6 h before irradiation, does administration of the erythrocytes at this time, in combination with the calcium antagonists improve on the radiosensitisation seen with transfusion immediately prior to X-rays? The answer given in Figure 5 is that it does not. This may be expected, since maximal radiosensitisation by the erythrocyte transfusion at this time interval suggests that the transfused red cells have reached their optimal state of oxygenation, and that the tumour has adapted to the increased viscosity produced by the additional red cells. Thus the administration of flunarizine at the same time as or after the erythrocyte transfusion may not be expected to improve on the radiosensitising ability of the transfusion itself. The cell survival levels for the administration of flunarizine 4 h before X-rays, whether transfusion is given 0 h or 4 h before irradiation, are not significantly different ($P>0.1$). However, it may be of interest to determine whether giving flunarizine at even earlier times before the erythrocyte transfusion, that is more than 4 h before X-rays, can improve further the radiosensitisation by the combination treatment. This may be possible, since Figure 4 suggests that the administration of flunarizine to anaemic mice appears to increase the optimal time of radiosensitisation to at least 4 h.

The results presented indicate that some extra benefit in X-ray sensitivity may be obtained by combining methods of increasing oxygen delivery to tumours, when the methods target different types of hypoxic cell. In the case of the SCCVII/St tumour, the combined radiosensitisation may be alternatively described as an improvement in the effects of the erythrocyte transfusion by the calcium antagonists, in addition to the direct effects of the agents themselves.

In terms of the clinical relevance of the results presented, there is no doubt that this type of treatment combination can greatly improve radiation sensitivity. For example, the administration of a blood transfusion to previously anaemic cancer patients may have some benefit, indicated in clinical trials for cancer of the cervix and head and neck (Bush *et al.*, 1978; Overgaard *et al.*, 1986). However, the recent concern over contamination of blood products suggests that the search for an alternative to this method of tumour oxygenation would be advantageous. These may include the use of hyperbaric oxygen or carbogen, where the increased tumour perfusion induced by the calcium antagonists would complement the increased oxygen carrying capacity of the blood. The adminstration of perfluorocarbons with calcium antagonists may be a promising combination, again with the improved tumour perfusion allowing further passage of the oxygen carrying perfluorocarbon molecules to remote tissues. Finally, the use of agents which reduce haemoglobin affinity for oxygen, such as the antilipidaemic agents, clofibrate and bezafibrate have been shown to increase tumour radiosensitivity (Hirst & Wood, 1988*a*) and might be expected to target the diffusion limited hypoxic cells within the tumour. These agents may therefore be expected to increase tumour radiosensitivity with calcium antagonists. However, preliminary experiments with clofibrate and flunarizine in this laboratory produce a less than additive radiosensitisation, which may be due to clofibrate also exhibiting the ability to increase tumour perfusion (Hirst & Wood, 1988*b*).

In conclusion, the use of the calcium antagonists cinnarizine and flunarizine to improve the radiosensitising ability of erythrocyte transfusion to anaemic mice is demonstrated, and although blood transfusion is not considered a treatment of choice as an adjuvant to radiotherapy, the calcium antagonists may have a future in combination with other, established methods of altering tumour oxygen delivery.

Supported by grant CA25990 from the United States National Cancer Institute.

### References

BROWN, J.M. (1979). Evidence for acutely hypoxic cells in mouse tumours, and a possible mechanism of reoxygenation. *Br. J. Radiol.*, **52**, 650.

BUSH, R.S., JENKIN, R.D.T., ALLT, W.E.C. & 4 others (1978). Definitive evidence for hypoxic cells influencing cure in cancer therapy. *Br. J. Cancer*, **37**, suppl. III, 302.

CHAPLIN, D.J., DURAND, R.E. & OLIVE, P.L. (1986). Acute hypoxia in tumours: implications for modifiers of radiation effects. *Int. J. Radiat. Oncol. Biol. Phys.*, **12**, 1279.

DECREE, J., DECOCK, W., GEUKINS, H., DECLERCK, F., BEERENS, M. & VERHAEGEN, H. (1979). The rheological effects of cinnarizine and flunarizine in normal pathological conditions. *Angiology*, **30**, 505.

HILL, R.P. & STIRLING, D. (1987). Oxygen delivery and tumour response. In *Radiation Research (Proceedings of 8th International Congress of Radiation Research, Edinburgh, July, 1987)*, Fielden, E.M., Fowler, J.F., Hendry, J.H. & Scott, D. (eds) p. 725. Taylor & Francis.

HIRST, D.G., BROWN, J.M. & HAZLEHURST, J.L. (1982). Enhancement of CCNU cytotoxicity by misonidazole: studies of possible therapeutic gain. *Br. J. Cancer*, **46**, 109.

HIRST, D.G., HAZLEHURST, J.L. & BROWN, J.M. (1984). The effects of alterations in haematocrit on tumour sensitivity to X-rays. *Int. J. Radiat. Biol.*, **46**, 345.

HIRST, D.G. & WOOD, P.J. (1987). The adaptive response of mouse tumours to anaemia and retransfusion. *Int. J. Radiat. Biol.*, **51**, 597.

HIRST, D.G. & WOOD, P.J. (1988*a*). Altered radiosensitivity in a mouse carcinoma after administration of clofibrate and bezafibrate. *Eur. J. Cancer Clin. Oncol.* (in the press).

HIRST, D.G. & WOOD, P.J. (1988*b*). Tumour radiosensitisation by clofibrate and its analogues: possible mechanisms. *Int. J. Radiat. Biol.*

JIRTLE, R.L. (1988). Chemical modification of tumour blood flow. *Int. J. Hyperthermia*, **4**, 355.

KAELIN, W.G., SHRIVASTAV, S., SHAND, D.G. & JIRTLE, R.L. (1982). Effect of verapamil on malignant tissue blood flow in SMT-2A tumour bearing rats. *Cancer Res.*, **42**, 3944.

KAELIN, W.G., SHRIVASTAV, S. & JIRTLE, R.L. (1984). Blood flow to primary tumours and lymph node metastases in SMT-2A tumour-bearing rats following intravenous flunarizine. *Cancer Res.*, **44**, 896.

OVERGAARD, J., SAND HANSEN, H., JORGENSEN, K. & HJELM HANSEN, M. (1986). Primary radiotherapy of larynx and pharynx carcinoma–an analysis of some factors influencing local control and survival. *Int. J. Radiat. Oncol. Biol. Phys.* **12**, 515.

TWENTYMAN, P.R., BROWN, J.M., GRAY, J.W., FRANKO, A.J., SCOLES, M.A. & KALLMAN, R.F. (1980). A new mouse tumour model system (RIF-1) for comparison of end-point studies. *J. Natl Cancer Inst.*, **64**, 595.

WOOD, P.J. & HIRST, D.G. (1988). Cinnarizine and flunarizine as radiation sensitisers in two murine tumours. *Br. J. Cancer*, **58**, 742.